# Treatment of peroneal nerve injuries with simultaneous tendon transfer and nerve exploration

**DOI:** 10.1186/s13018-014-0067-6

**Published:** 2014-08-06

**Authors:** Bryant Ho, Zubair Khan, Paul J Switaj, George Ochenjele, Daniel Fuchs, William Dahl, Paul Cederna, Theodore A Kung, Anish R Kadakia

**Affiliations:** 1 Department of Orthopaedic Surgery, Feinberg School of Medicine, Northwestern University, 676 N. St. Clair, Suite 1350, Chicago 60611, IL, USA; 2 Department of Orthopaedic Surgery, University of Michigan, 2912 Taubman Center, 1500 E. Medical Center Dr, Ann Arbor 48109, MI, USA; 3 Department of Plastic Surgery, University of Michigan, 2130 Taubman Center, 1500 E. Medical Center Dr, Ann Arbor 48109, MI, USA

**Keywords:** Common peroneal nerve palsy, Peroneal nerve palsy, Common peroneal nerve, Peroneal nerve injury, Foot drop, Drop foot, Posterior tibialis, Tibialis posterior, Flexor digitorum longus, Tendon transfer

## Abstract

**Background:**

Common peroneal nerve palsy leading to foot drop is difficult to manage and has historically been treated with extended bracing with expectant waiting for return of nerve function. Peroneal nerve exploration has traditionally been avoided except in cases of known traumatic or iatrogenic injury, with tendon transfers being performed in a delayed fashion after exhausting conservative treatment. We present a new strategy for management of foot drop with nerve exploration and concomitant tendon transfer.

**Method:**

We retrospectively reviewed a series of 12 patients with peroneal nerve palsies that were treated with tendon transfer from 2005 to 2011. Of these patients, seven were treated with simultaneous peroneal nerve exploration and repair at the time of tendon transfer.

**Results:**

Patients with both nerve repair and tendon transfer had superior functional results with active dorsiflexion in all patients, compared to dorsiflexion in 40% of patients treated with tendon transfers alone. Additionally, 57% of patients treated with nerve repair and tendon transfer were able to achieve enough function to return to running, compared to 20% in patients with tendon transfer alone. No patient had full return of native motor function resulting in excessive dorsiflexion strength.

**Conclusion:**

The results of our limited case series for this rare condition indicate that simultaneous nerve repair and tendon transfer showed no detrimental results and may provide improved function over tendon transfer alone.

## Introduction

Common peroneal nerve palsy is the most common mononeuropathy of the lower extremity and may resolve spontaneously. However, irreversible nerve damage can occur, with historically poor outcomes [1],[2]. Patients present with dorsal foot sensory loss, as well as loss of ankle dorsiflexion from the tibialis anterior and loss of foot eversion from the peroneus longus and brevis. The unopposed pull of the tibialis posterior and Achilles results in an equinovarus foot deformity, while the loss of the foot dorsiflexors results in a foot drop, with characteristic foot slap during heel strike and a steppage gait [3].

Most peroneal nerve injuries are iatrogenic or secondary to trauma [4]. Iatrogenic causes include complications of total knee replacements, while traumatic causes include lacerations, hip or knee dislocations, and fractures [5]-[7]. Peroneal nerve palsies can also occur from local compression by tumors, ganglion cysts, or poor positioning in the operative and immediate postoperative periods [8]-[11]. Other rare reported causes include ankle sprains, acupuncture, or crural vein thrombosis [12]-[14].

Traditional treatment in patients with no clear etiology of nerve injury includes symptomatic management with physical therapy and an ankle-foot orthosis (AFO) to prevent equinovarus deformity. Observation is advocated for a minimum of 6 months, and often longer, as many of the previously reported peroneal nerve palsies recovered or had minimal residual deficits [15],[16]. In patients without a known injury or causally related factor, we agree with expectant observation as historically described.

Operative intervention has been advocated for all etiologies 2 years from onset in patients who have not recovered function and do not tolerate the use of an AFO [17]. The main reported techniques for operative intervention are summarized in Table 1. Treatment options involve two main strategies: restoration of peroneal nerve function and tendon transfer to restore muscle function and balance of the foot. Peroneal nerve interventions include neurolysis, neuroplasty, or cable graft nerve repair. A branch of the tibial nerve can also be transferred to the distal deep peroneal nerve, known as a nerve transfer.

**Table 1:** Techniques for treatment of peroneal nerve palsy

**Author**	**Technique**	**Results**
Tendon transfer		
Watkins et al. [18]	PTT transfer through IO to lateral cuneiform	17/29 patients with excellent results, 7 good, 1 fair, 4 unknown^a^
Prahinski et al. [19]	PTT transfer through IO to triple anastomosis between PTT, ATT, and PL (Bridle procedure)	6/10 patients brace free, 10/10 satisfied
Wagenaar and Louwerens [20]	PTT transfer through IO to extensor tendons proximal to ankle	10/11 patients brace free, 10/11 with good/fair gait
Pinzur et al. [21]	ATT and PTT transfer to lateral cuneiform	9/9 patients brace free with subjective improvement
Nerve repair + tendon transfer		
Ferraresi et al. [22]	PTT transfer through IO to lateral cuneiform; peroneal nerve repair with sural nerve graft	48/53 patients with nerve regeneration, 28/53 good result, 10 fair, 6 poor
Nerve transfer		
Nath et al. [23]	Tibial nerve transfer to deep peroneal nerve	12/14 patients with strength improvement, 10/14 dorsiflexion strength ≥4
Ninkovic and Ninkovic [24]	Gastrocnemius transfer with reinnervation to the undamaged proximal peroneal nerve	18/18 patients brace free with gait improvement and voluntary dorsiflexion, 10/18 excellent results, 4 good, 3 satisfactory, 1 poor

*PTT* posterior tibial tendon, *IO* interosseous membrane, *ATT* anterior tibial tendon, *PL* peroneus longus. ^a^Reported results in muscular imbalance patients.

Tendon transfers partially restore function of the foot and most commonly the posterior tibialis tendon (PTT) is transferred to the lateral cuneiform or cuboid to restore ankle dorsiflexion. This transfer has the added benefit of removing the primary deforming force of the foot. Additionally, based on the preoperative exam for equinus, a tendo-Achilles lengthening or gastrocnemius recession (GSR) may be required.

Both peroneal nerve repair and PTT transfer have efficacy in treating patients with peroneal nerve palsy [25],[26]. However, Garozzo et al. showed superior results in patients treated with both peroneal nerve repair and PTT transfer when compared to isolated nerve repair with dorsiflexion strength against gravity in 58% of patients in the former group compared to only 22% in the latter [27].

We present our experience treating peroneal nerve palsies, comparing treatment with simultaneous nerve repair and PTT transfer versus treatment with isolated tendon transfer in patients with an identifiable injury to the peroneal nerve.

## Materials and methods

### Patient population

This is a retrospective series of 12 consecutive patients with peroneal nerve palsy that were treated between 2005 and 2011. There were ten males and two females, age 19–59 years with a mean age of 38 (Table 2). Six patients had peroneal nerve palsy from a knee dislocation, two from a nerve laceration, three from iatrogenic injury during an open procedure, and one from a posterior wall acetabulum fracture (Table 3). All patients had complete preoperative dorsiflexion motor deficit, and electromyogram (EMG) results on nine patients showed complete absence of common peroneal nerve function, while the EMG results of three patients showed abnormal residual function (Table 3). All patients with clinical foot drop were offered surgical intervention unless EMG showed normal function. All patients had a PTT transfer to the lateral cuneiform. This was the sole procedure in the first five consecutive patients treated in this study from 2005 to 2007, termed the control group.

**Table 2:** Demographics

**Characteristic**	**Control**	**Concomitant**
Mean age (years ± SD)	43 ± 13	37 ± 13
Age range (years)	30–60	21–53
Gender		
Male	5 (100%)	5 (71%)
Female	0 (0%)	2 (29%)

**Table 3:** Patient data and interventions

	**Injury**	**Time to surgery (months)**	**EMG**	**Nerve intervention**	**Tendon transfer**	**Maximum activity**
1	Knee dislocation	12.7	+^a^	Neurolysis	PTT, FDLT	Walk
2	Knee dislocation	6.2	−	Neurolysis	PTT, FDLT, GSR	Run
3	Knee dislocation	9.1	−	Neuroplasty	PTT, FDLT	Walk
4	Knee dislocation	27.8	−	None	PTT, TAL	Walk
5	Posterior wall acetabulum fracture	25.5	−	None	PTT, FDLT, GSR	Walk
6	Iatrogenic, tumor resection	49.4	−	Sural nerve graft 6-cm defect^b^	PTT, GSR	Run
7	Nerve laceration	13.7	+	Neurolysis	PTT, FDLT	Run
8	Knee dislocation	13.6	+	None	PTT, TAL	Walk
9	Laceration	7.7	−	Sural nerve graft 4.5-cm defect	PTT, FDLT	Run
10	Iatrogenic, tumor resection	31	−	None	PTT, TAL	Run
11	Iatrogenic, tibial plateau ORIF	27.9	−	None	PTT, TAL	Walk (AFO)
12	Knee dislocation	9.5	−	Neurolysis	PTT, TAL, FDLT	Walk

*PTT* posterior tibial transfer, *FDLT* flexor digitorum longus transfer, *GSR* gastrocnemius recession, *TAL* tendo-Achilles lengthening, *ORIF* open reduction internal fixation, *AFO* ankle foot orthosis. ^a^ + EMG refers to positive but abnormal function; ^b^nerve graft 2 years prior to tendon transfer.

Review of the literature in 2007 led us to subsequently change our surgical protocol. Seven consecutive patients were treated from 2007 to 2011 with PTT tendon transfer and nerve repair performed by a plastic surgeon, termed the concomitant group. All tendon transfers and nerve repairs were done simultaneously except for one patient who had a sural nerve graft 2 years prior to PTT tendon transfer. Of these seven patients in the concomitant group, four patients had neurolysis, one patient had a neuroplasty, and two patients had sural nerve grafting.

Intervention to lengthen the gastroc/soleus complex was considered if the patient did not have 5° of dorsiflexion with the knee extended. If the patient had an isolated gastrocnemius contracture, an isolated gastrocnemius recession was performed. Of the control group, one patient had a GSR, and four patients had a TAL. Of the concomitant group, two patients had a GSR, and one patient had a TAL.

The patients in the control group underwent operative intervention at a mean of 25 months (range 13–31) after diagnosis of injury with postoperative follow-up for a mean of 17 months (range 3–27). The patients in the concomitant group underwent operative intervention at a mean of 15.5 months (range 6–49) after diagnosis of injury with postoperative follow-up for an average of 24 months (range 12–48).

At the final follow-up, a motor strength was evaluated on physical exam on a scale of 0–5. Sensation was evaluated to light touch on in all lower extremity nerve distributions. Pain was assessed as a yes or no answer with further questioning on specific nature and distribution of the pain. Functional level was assessed through questioning on use of ambulatory aids as well as activity level and participation in sports. Patient satisfaction was assessed on a scale of extremely satisfied, satisfied, satisfied with reservation, and dissatisfied.

### Surgical protocol

**Figure 1:**
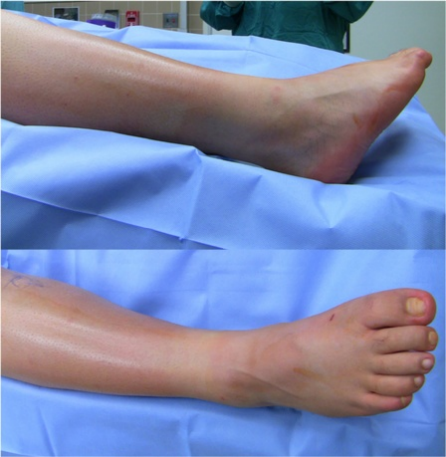
**Preoperative positioning for a patient who required an isolated tendon transfer.** Note the preoperative equinovarus and the neutral rotation of the foot that facilitates access to the medial and lateral aspects of the leg.

**Figure 2:**
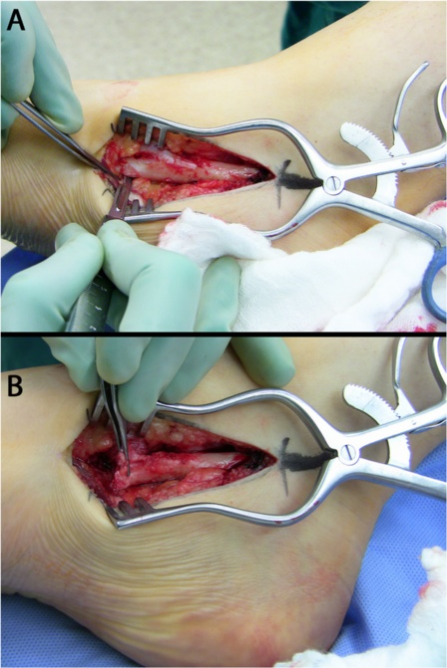
**A medial incision is made over the PTT (A), and the PTT is harvested subperiosteally (B).** The PTT is harvested subperiosteally from the navicular beginning at the naviculo-cuneiform joint to maximize tendon length.

The patient is positioned supine with a bump under the ipsilateral hip to internally rotate the affected leg 45° in the cases of a combined nerve and tendon transfer. In cases of isolated tendon transfer, the hip is bumped to create a neutral position of the foot such that the toes pointed to the ceiling (Figure 1). The PTT transfer is performed through a posteromedial incision over the PTT just distal to the medial malleolus, extending approximately 5 cm distally. The tendinous insertion on the navicular is dissected subperiosteally and released from distal (naviculo-cuneiform joint) to proximal to preserve length (Figure 2). Care must be taken not to transect the tendon at the navicular insertion to avoid having insufficient tendon length.

**Figure 3:**
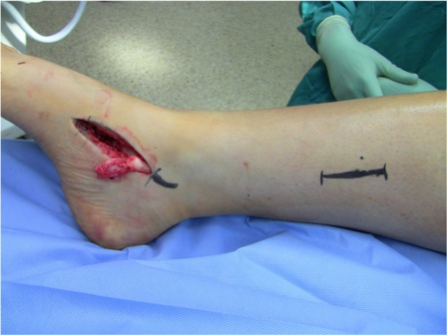
The second incision is made 15 cm proximal to the tip of the medial malleolus.

**Figure 4:**
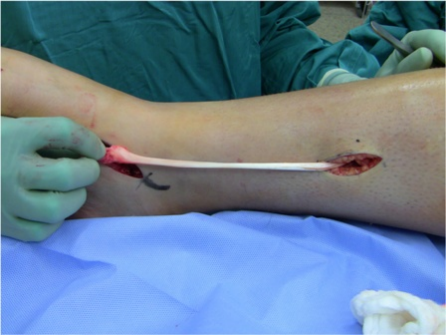
The PTT is delivered into the wound with careful attention not to entrap the neurovascular bundle.

A second 3-cm incision is made approximately 15 cm proximal to the tip of the medial malleolus (Figure 3). The saphenous vein and nerve are dissected and retracted anteriorly to expose the fascia of the deep posterior compartment. The soleus and flexor digitorum longus (FDL) are retracted posteriorly, and the PTT is identified adjacent to the tibia and the interosseous membrane and confirmed with countertraction to ensure that the neurovascular bundle lying posteriorly is spared. The PTT is delivered through the proximal incision with either a right angle hemostat or digital manipulation and tagged with a number 0 FiberWire (Arthex, Naples, FL, USA) with a locking whipstitch to facilitate transfer (Figure 4).

**Figure 5:**
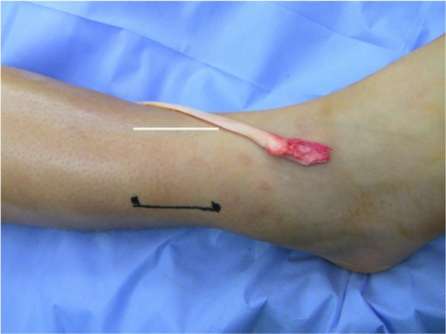
**PTT placed laterally over the leg to locate optimal path through the interosseous membrane (*white line*).** A 5-cm lateral incision is then placed directly lateral to this *line* over the anterior border of the fibula.

**Figure 6:**
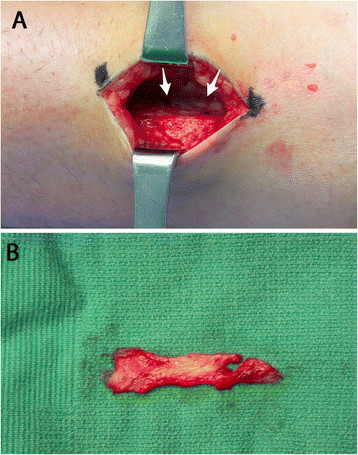
**Visualization and excision of the interosseous membrane. (A)** The interosseous membrane (*white arrows*) is visualized after the extensor digitorum longus is elevated medially. **(B)** A 4-cm length of the interosseous membrane is excised to allow for free passage of the PTT without entrapment or scarring.

A 5-cm incision is placed along the anterior border of the fibula. The distal aspect of this incision is determined by placing the PTT over the anterolateral aspect of the leg to the distal fibula to find the approximate intersection between the PTT and the interosseous membrane (Figure 5). Approximately 4 cm of the interosseous membrane is dissected off the fibula and excised to allow free passage of the PTT without risk of entrapment of the muscle belly or scarring of the tendon (Figure 6). Care must be taken to avoid damage to the neurovascular bundle as this now lies directly adjacent to the interosseous membrane after delivery of the PTT through the medial wound. The PTT is then passed from the proximal incision through the distal lateral incision by sliding the tendon against the posterior tibia to avoid the neurovascular bundle.

**Figure 7:**
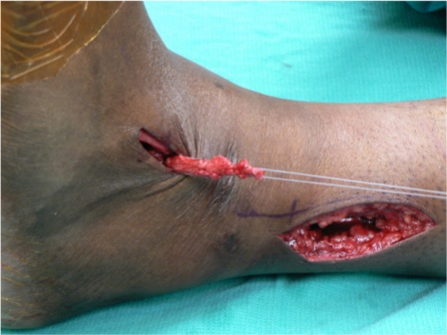
**Passage of the PTT from the medial to lateral wound via the interosseous membrane window.** The clamp must slide directly against the posterior tibia to avoid damage to the neurovascular bundle. The tendon is then taken subcutaneously to a dorsal incision over the lateral cuneiform.

A 2-cm longitudinal incision is made over the dorsal surface of foot overlying the lateral cuneiform after fluoroscopic confirmation. The PTT is then passed subcutaneously from the distal lateral incision through the dorsal foot incision (Figure 7). We prefer the subcutaneous position to maximize the lever arm of the transferred tendon to improve power while compromising the overall excursion.

**Figure 8:**
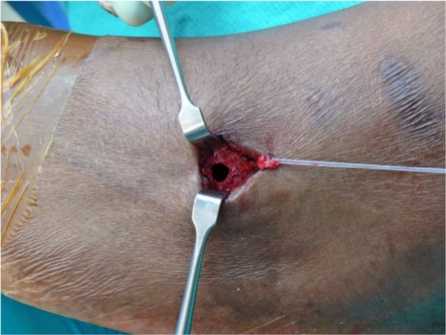
**A drill hole is placed in the lateral cuneiform under fluoroscopy.** The size of the hole is typically 5 mm; however, it may need to be modified based on the size of the tendon.

**Figure 9:**
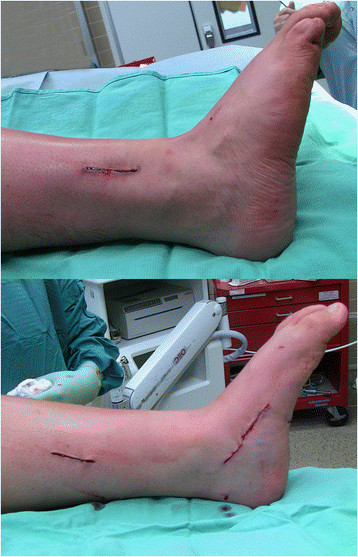
**Final positioning at 5° equinus in a concomitant group patient.** This prevents a tenodesis effect and provides for adequate plantarflexion for running.

A 5-mm drill hole is then placed in the lateral cuneiform (Figure 8). Tensioning of the tendon was altered for patients who underwent simultaneous nerve and tendon reconstruction. Our goal was to achieve the ability to run, and therefore, it was felt that creating a tenodesis effect by maximal tension would be inappropriate in this select group. Therefore, the PTT was placed under maximal tension with the foot held in 5° of plantarflexion. The PTT is then relaxed by 1 mm and fixed with a 6.25-mm interference screw (Arthex) so that the final fixation places the foot in slight plantarflexion in cases where a concomitant nerve reconstruction was performed (Figure 9). This positioning of the foot ensures adequate postoperative plantarflexion, which is critical to provide enough mechanical advantage to allow high-performance activities such as running. However, this clearly risks persistent foot drop if the tendon transfer fails to achieve active function. In cases that presented in a delayed fashion that were not candidates for a nerve reconstruction, the tendon was maximally tensioned at neutral.

All FDL tendon transfers were simultaneously performed with fixation performed through a drill hole in the navicular. Given the lack of distal posterior tibial tendon, the FDL is passed from plantar to dorsal via a 4.5-mm drill hole. The tendon is tensioned with the foot held in neutral inversion to avoid excess strain on the posterior tibial tendon transfer. Fixation is performed with multiple figure of eight nonabsorbable sutures to itself and to the periosteum.

**Figure 10:**
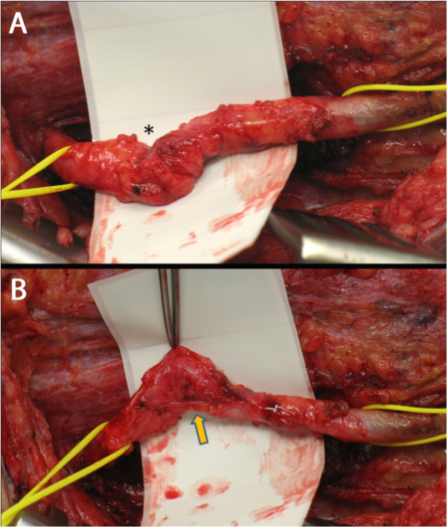
**Peroneal nerve exploration. (A)** Right common peroneal nerve found to have a localized area of scarring (*asterisk*). **(B)** During neurolysis, deforming scar bands (*arrow*) were identified and released, revealing a completely intact common peroneal nerve. This finding obviated the need for nerve resection and cable grafting.

At the completion of tendon transfers, the peroneal nerve is explored. An incision is made lateral to the knee along the course of the peroneal nerve, extending distally between the lateral and anterior compartments of the lower leg. Dissection proceeds over the fibular head, and the investing fascia over the common peroneal nerve is sharply divided. The course of the nerve is identified proximally and distally as it divides into superficial and deep branches. Complete neurolysis is then performed over the fully exposed peroneal nerve (Figure 10).

The peroneal nerve is carefully inspected for evidence of transection or neuroma formation. A neuroma-in-continuity is identified by the presence of a localized area of swelling within an otherwise intact nerve. Neurolysis is completed by resection of all surrounding scar tissue in order to ensure free mobility of the nerve. The nerve itself is then inspected and if found to be intact, no further interventions are performed and the wound is closed in layers. However, if continuity of the nerve is disrupted (partial or complete) or if a neuroma-in-continuity is recognized, the involved portion of the nerve is resected, and the resultant gap is addressed with sural nerve cable grafting. It is essential to perform adequate resection until healthy-appearing nerve tissue is encountered proximally and distally.

The required length of sural nerve for cable grafting of the defect is measured. After inflating a thigh tourniquet, a 5-cm longitudinal incision is made between the lateral malleolus and the Achilles tendon. The sural nerve is found adjacent to the lesser saphenous vein and is meticulously dissected free. The incision is extended proximally as necessary, following the course of the sural nerve between the two heads of the gastrocnemius muscle. The sural nerve graft is harvested, reversed in its orientation, and divided into segments for interpositional cable grafting of the common peroneal nerve and its superficial and deep branches. Coaptations are performed under the operating microscope using 9-0 interrupted nylon epineurial sutures. After wound closure, a plaster posterior splint is fabricated to maintain the ankle in dorsiflexion, and a knee immobilizer is utilized to prevent tension on the nerve reconstruction for the first 2 weeks.

The patients are kept nonweight-bearing in a splint for 2 weeks, after which they are switched to a weight bearing cast for 4 weeks. At 6 weeks postoperatively, they are transitioned to a CAM boot, and physical therapy is begun without passive plantarflexion. At 3 months, activity is advanced as tolerated with the exception of impact activities, which begin at 6 months. The patients are kept in an AFO at night for the first 3 months.

## Results

In the control group, three patients were extremely satisfied, one patient was satisfied, and one patient was satisfied with reservation (Table 4). In the concomitant group, six patients were extremely satisfied, and 1 patient was dissatisfied. The patient who was dissatisfied had concomitant compartment syndrome and traumatic tibiotalar arthritis with chronic pain that additionally affected the outcome.

**Table 4:** Results comparing tendon transfer with nerve repair (concomitant) to isolated tendon transfer (control)

	**Patients**	**Pain**	**Outcome**				**DF intact**	**Activity**	
			**ES**	**S**	**SR**	**D**		**Walk**	**Run**
Concomitant	7	1 (14%)	6 (86%)	0	0	1 (14%)	7 (100%)	7 (100%)	4 (57%)
Control	5	2 (40%)	3 (60%)	1 (20%)	1 (20%)	0	2 (40%)	4 (80%)	1 (20%)

*ES* extremely satisfied, *S* satisfied, *SR* satisfied with reservations, *D* dissatisfied, *DF* dorsiflexion.

Two patients in the control group reported pain on the final follow-up associated with preexisting chronic neuropathy and preexisting complex regional pain syndrome, respectively. One patient in the concomitant group reported pain associated with previous compartment syndrome and traumatic tibiotalar arthritis. No patients reported pain related to operative intervention.

In the control group, two (40%) patients achieved active dorsiflexion with strength of 3 and 4, respectively, on a scale of 5. Three patients (60%) had 0/5 strength. In the concomitant group, all patients had active dorsiflexion. One patient had 1/5 strength, and one patient had 3/5. Three patients had 4/5 strength, and two patients had 5/5 (Additional file 1: Video 1).

Preoperatively, all patients in the control group lacked sensation in the superficial peroneal nerve (SPN) and the deep peroneal nerve (DPN) distributions. Postoperatively, no patients noted a change in their sensory function. In the concomitant group, all patients lacked SPN and DPN sensation preoperatively except for the patient who had a prior sural nerve graft 2 years prior who had intact but diminished sensation. Postoperatively, three (43%) reported intact sensation, two (29%) reported intact but diminished sensation, and one (14%) reported no sensation. The prior sural nerve graft patient had no change in sensation.

In the control group, maximum achieved activity consisted of running in one patient (20%), three patients (60%) were able to walk without assistive devices, and one patient (20%) required an AFO (Additional file 2: Video 2). In the concomitant group, all patients were able to walk without assistive devices. Four patients (57%) were able to run, and three patients (43%) were only able to walk without assistive devices (Additional file 3: Video 3).

All patients in the control group had a preoperative equinus contracture requiring a GSR in one patient (20%) and a TAL in four patients (80%). Only three patients (43%) in the concomitant group had an equinus contracture requiring GSR in two patients (29%) and TAL in one patient (14%). There were no complications related to the procedures at the time of the final follow-up.

## Discussion

Common peroneal nerve palsy can occur through a variety of mechanisms, although the exact etiology is often unknown. The superficial location of the nerve as it courses around the fibular neck exposes the nerve to injury from penetrating and blunt trauma, or from external compression, and the decreased blood supply to the common peroneal nerve compared to the tibial nerve [28] may explain the increased susceptibility to injury and poor results with nerve repair. Nobel suggested that tensile forces associated with lower extremity trauma could result in tears of the nutrient arteries supplying the common peroneal nerve [29]. Additionally, Lundborg and Rydevik found that 8% elongation of nerves resulted in impaired microcirculation, while 15% elongation resulted in complete nerve ischemia in rabbits [30].

Peroneal nerve palsies are typically neuropraxic nerve injuries where an intact nerve sheath guides rapid reinnervation, typically in 3–4 weeks. Crush- or contusion-type injuries typically result in axonotmesis nerve injuries, in which Wallerian degeneration of the axon occurs, which must be regenerated at a time-dependent rate of 1–2 mm/day. In the common peroneal nerve, the length of the nerve poses a challenge to reinnervation. In traumatic lacerations, the nerve should be immediately repaired. However, in neuropraxic injuries of unclear origin, there is a high probability of nerve function return.

Recovery after common peroneal nerve injury is diminished when compared to other lower extremity nerve injuries. More nerve fibers are needed to provide clinical dorsiflexion, as opposed to the bulky gastrocnemius complex, in which little innervation is needed to provide clinical plantarflexion. The extensive branching of the deep peroneal nerve leads to varying rates of reinnervation of the anterior compartment. This subsequently causes uncoordinated contraction and decreased dorsiflexion force [31].

Nerve conduction studies should be performed 12 weeks after injury if peroneal palsy persists, as the value of EMG is limited prior to this time [32]. If EMG is abnormal, we believe surgical intervention should be considered at this time given that our clinical experience has shown a lack of nerve regeneration after 3 months. We understand a limitation in our study is the variable in the delay of presentation and time to intervention. However, we are unable to control for this factor, as the majority of our patients did not initially present to our institution for their injury.

Once a diagnosis has been made, there are two main goals of treatment: establishment of nerve function and restoration of muscle balance and function. Early peripheral nerve repair has been shown to yield superior outcomes over delayed repair [33]. Studies have shown that regeneration of axons and reinnervation of muscle fibers declines in a time-dependent fashion [34],[35]. Seidel et al. showed good results in patients treated with early neurolysis for peroneal nerve palsies, with 72% of patients achieving motor strength four out of five or greater [25]. However, the likelihood of full return of motor function is low, and 28% of patients did not achieve 4/5 strength with isolated nerve reconstruction. Thus, our protocol adds the use of early simultaneous tendon transfer to maximize postoperative function.

The use of tendon transfers for persistent palsies have traditionally been considered only in select cases, and often occurring in a delayed fashion from than a year from the initial injury. If nerve repair was performed, tendon transfers were delayed for up to 2 years after to allow sufficient time for recovery of possible nerve function [17]. In contrast, early tendon transfers in the upper extremity have historically been widely accepted for ulnar and radial nerve palsies [36],[37]. Tendon transfers for radial nerve palsies provided immediate function while serving as an internal splint to prevent flexion contractures or need for external splinting.

One disadvantage of delayed tendon transfer is that many of these patients develop rigid equinus contractures that require intervention. Although an Achilles lengthening will restore the functional motion, it cannot restore the normal length-tension relationship of the gastroc-soleus complex, which will result in a permanent functional deficit in plantarflexion. Early intervention that does not allow a rigid equinus contracture to develop allows the patient to maintain plantarflexion power and may account for the relatively high incidence of running and athletic activity that we have reported. Another drawback of delayed transfers is that the use of an AFO for 2 years may results in disuse atrophy of the posterior tibial tendon. The transfer of a muscle that has atrophied over the course of 2 years clearly will limit the function of the transfer. We feel that the transfer of the muscle prior to significant atrophy will improve the functional results. Unfortunately, we cannot identify this factor in isolation in our study secondary to the concomitant nerve reconstructions that were performed.

Nerve function is improved when early nerve repair is combined with tendon transfer. Ferraresi et al. found nerve regeneration in 90% of patients who had nerve repair in conjunction with tendon transfers compared to nerve regeneration in only 17% of patients who had only nerve repair alone [22]. Similarly, Burkhalter found marked early and late functional improvement in patients with radial nerve palsy who had nerve repair with early tendon transfer [38]. We found similar results in our study, with superior results in patients who had nerve repair and tendon transfer. Fifty-seven percent of these patients (4/7) were able to run, with two patients returning to competitive sports. Dorsiflexion was intact in all patients with nerve repair and tendon transfer compared to dorsiflexion in only 40% of patients with tendon transfers alone.

While the source of this benefit is unclear, we hypothesize that the maintenance of foot balance and active dorsiflexion with tendon transfers creates a favorable environment for nerve regeneration. Prolonged denervation of muscle results in decreased reinnervation capacity of the injured nerve. This is not due to fat atrophy of the muscle, but instead to an intrinsic progressive loss of the capacity for axons to regenerate [39]. Specific neurotrophic factors that promote neuron growth are upregulated in Schwann cells of damaged nerves. However, this gene expression progressively declines in chronic Schwann cell denervation [40]. We believe that early tendon transfers may provide their positive influence on nerve regeneration by stimulating neurotrophic factors in damaged nerves that increase axon regeneration potential.

An additional benefit to early tendon transfer is that patients have immediate active dorsiflexion and can typically resume unassisted ambulation at 3 months postoperatively. By providing an internal tendinous splint that prevents equinus contracture, patients avoid transient or permanent loss of plantarflexion strength associated with Achilles lengthening procedures [41],[42].

We believe that the high level of function reported in our patients was due not only to our aggressive early combined approach but also to aspects of our surgical technique. Previous methods of PTT transfer to the dorsal foot have been limited by insufficient tendon length [18]. In our protocol, we reattach the PTT to the lateral cuneiform to increase tendon length while maintaining neutral supination and pronation of the foot [43]. The PTT is routed superficial to the extensor retinaculum to increase dorsiflexion efficiency by increasing the moment arm [44]. We tension the PTT in slight plantarflexion to establish maximum plantarflexion excursion and optimal muscle tension which allows the generation of the maximal plantarflexion force that is critical in high-level activities such as running. Maas and Huijing have shown in rats that tendon transfers that are overlengthened can decrease the strength of the remaining previously synergistic muscles of the transferred tendon [45]. By avoiding this overlengthening, we aim to decrease possible subsequent decrease in plantarflexion strength. We also transfer the FDL to the navicular in order to protect against the theoretical risk of an iatrogenic flatfoot deformity caused by PTT transfer, although no data exists to support this and only one case has been reported in the literature [46].

Using these methods, our results were uniformly excellent aside from one specific case with unique circumstances. This patient had antecedent post-traumatic arthritis and underwent both nerve repair and tendon transfer with the understanding of low likelihood of pain resolution and high likelihood of needing a future ankle fusion. Following the operation, this patient continued to have high levels of pain and dissatisfaction, most likely secondary to her pre-existing condition.

If this patient is excluded from our series, then all patients who had combined nerve repair and tendon transfer had excellent outcomes, with high satisfaction and without postoperative pain at final follow-up. Four of five patients (80%) achieved ankle dorsiflexion strength of 4/5, while one patient had strength of 3/5. Additionally, 66% of the patients were able to run, and 33% of these patients were able to return to competitive sports. To our knowledge, this is the first report of peroneal nerve palsy patients achieving such high postoperative function. While Nath et al. showed similarly successful results in peroneal palsy patients treated with nerve transfer with 10/14 (71%) patients achieving 4/5 ankle dorsiflexion strength, there are no reports of high-end function such as running or sports [23]. The morbidity of a tibial nerve transfer includes a 5% to 10% reduction in calf circumference from denervation of the lateral gastrocnemius muscle, a possible contributor to plantarflexion weakness that may limit strenuous activity.

While many of our patients had excellent results, there was a trend toward decreased function in patients with stretch-type injuries to the common peroneal nerve. Of the traumatic knee dislocation patients, only four (80%) were able to achieve maximum activity of walking. We believe that this is due to the mechanism of knee dislocations, which typically leads to a stretch injury of the nerve with a subsequent large zone of injury. Prasad showed on histologic examination that the zone of injury extends to the myoneural junction, with a distal zone of injury that is not visible intraoperatively, even with microscope magnification [47]. In this subset of patients, a nerve transfer (neurotization) may be an alternative option to consider given the challenges to nerve repair. A nerve transfer allows a functioning nerve to be placed close to the myoneural junction to decrease the time-dependent regeneration of nerve. Giuffre et al. reported a dorsiflexion strength of 3/5 or greater in 36% of patients who had peroneal nerve palsy from high-energy knee injuries that underwent partial tibial nerve transfer to the tibialis anterior nerve branch [48]. When examining zone of injury, it is also important to note that four (80%) of our patients who were able to achieve maximum activity of running had well-defined, small zones of injury from iatrogenic or sharp trauma etiologies. This is likely the optimal candidate for nerve repair and PTT transfer.

Due to these encouraging results, we have adopted a protocol of simultaneous nerve repair combined with tendon transfer 3 months after injury if EMG confirms lack of reinnervation. In our experience, tendon transfers in patients with ankle pathology are contraindicated. Unless the ankle is additionally addressed, the patient is likely to have a poor outcome. Although our limited case series was not large enough to show any statistically significant findings, we found no detrimental results associated with nerve repair and tendon transfer. We believe that our positive results indicate that aggressive intervention should be considered in the management of patients with peroneal nerve palsy.

## Conclusion

The results of our limited case series indicate that simultaneous nerve repair and tendon transfer showed no detrimental results and may provide improved function over tendon transfer alone. Aggressive early intervention should be considered in patients with peroneal nerve palsies.

## Competing interests

The authors declare that they have no competing interests.

## Authors’ contributions

BH participated in the interpretation of the data and drafted the manuscript. ZK and WD enrolled patients in the study and gathered relevant data. GO, PS, and DF participated in drafting and editing of the manuscript. TK drafted the surgical protocol for nerve reconstruction. PC is a participating surgeon in the study and edited the surgical protocol for nerve reconstruction. AK is the lead surgeon of the study that he conceived and designed. All authors read and approved the final manuscript.

## Additional files

## Supplementary Material

Additional file 1: **Video 1.** Functional evaluation of a patient 18 months after right posterior tibialis tendon transfer and common peroneal nerve neurolysis. Note that the right foot demonstrates sufficient dorsiflexion to ambulate normally. Note the limited DF and PF compared to the contralateral normal foot. Click here for file

Additional file 2: **Video 2.** Postoperative gait of a patient who underwent isolated tendon transfer on the left lower extremity. Click here for file

Additional file 3: **Video 3.** Demonstration of running of the same patient in Additional file 1: Video 1 who underwent surgical correction of the right foot. Click here for file
